# ESolvent-free, enzyme-catalyzed biodiesel production from mango, neem, and shea oils via response surface methodology

**DOI:** 10.1186/s13568-015-0172-x

**Published:** 2015-12-23

**Authors:** Divine Bup Nde, Carlos Astete, Dorin Boldor

**Affiliations:** BAE Department, Louisiana State University Agricultural Center, Baton Rouge, LA 70803 USA; Department of Food and Bio-resource Technology, College of Technology, University of Bamenda, P.O. Box 39, Bamenda, Cameroon

**Keywords:** Biodiesel, Enzyme, Mango, Methyl esters, Neem, Shea

## Abstract

Mango, neem and shea kernels produce non-conventional oils whose potentials are not fully exploited. To give an added value to these oils, they were transesterified into biodiesel in a solvent-free system using immobilized enzyme lipozyme from *Mucor miehei*. The Doehlert experimental design was used to evaluate the methyl ester (ME) yields as influenced by enzyme concentration—EC, temperature—T, added water content—AWC, and reaction time—RT. Biodiesel yields were quantified by ^1^H NMR spectroscopy and subsequently modeled by a second order polynomial equation with interactions. Lipozyme enzymes were more tolerant to high temperatures in neem and shea oils reaction media compared to that of mango oil. The optimum reaction conditions EC, T, AWC, and RT assuring near complete conversion were as follows: mango oil 7.25 %, 36.6 °C, 10.9 %, 36.4 h; neem oil EC = 7.19 %, T = 45.7 °C, AWC = 8.43 %, RT = 25.08 h; and shea oil EC = 4.43 %, T = 45.65 °C, AWC = 6.21 % and RT = 25.08 h. Validation experiments of these optimum conditions gave ME yields of 98.1 ± 1.0, 98.5 ± 1.6 and 99.3 ± 0.4 % for mango, neem and shea oils, respectively, which all met ASTM biodiesel standards.

## Introduction

Over the last decades, the importance of biodiesel has grown steadily as its research moved solidly in the commercialization arena (Howell 2009). Among its advantages over conventional diesel fuels one can include the facts that biodiesel is biodegradable, renewable and produces low levels of CO_2_ which make it environmentally friendly fuel. However, biodiesel fuel does not compete favorably economically with conventional diesel due to the high cost of vegetable or animal oils, the principal raw material in biodiesel manufacture, and the lack of government subventions in most countries as it is the case with conventional diesel. Given the upward trends in the price of conventional diesel fuels and considering its exhaustive nature, it is feared that in the near future conventional diesel may become expensive and/or completely exhausted. There is therefore a critical need to search for suitable alternatives such as biodiesel to complement future fuel needs.

One feature of the transesterification reaction between the oils and short chain alcohol into biodiesel is the use of a catalyst to increase reaction rates and conversion yields. To this effect chemical catalysis and enzymatic catalysis have been used extensively in biodiesel research (Ma and Hanna 1999). Though the use of chemical catalysis in transesterification reactions is efficient in terms of reaction time, there are several setbacks such as difficulty in the recovery and purification of glycerol byproduct and the energy-intensive nature of the process, non-reusable nature of the recovered homogenous catalyst and soap formation (Sha et al. 2003). Enzymatic catalysis on its part is time consuming and the high cost of enzymes make the reaction very expensive. Enzymatic catalysis however has several advantages such as easy separation of by-products, synthesis of specific alky esters, and transesterification of glycerides with high free fatty acid content which eliminates the need for a two-step reaction process (Nelson et al. 1996). Several researchers have shown that the use of immobilized enzymes can significantly reduce biodiesel production cost because of the reusability of the immobilized enzymes (Dizge and Keskinler 2008; Shah and Gupta 2007). The use of enzymatic catalysis in biodiesel synthesis therefore remains a viable alternative.

So far, each country develops biodiesel feedstock according to its national conditions. For example, the United States mainly uses genetically modified soybean oil, the European Union and Canada use rapeseed oil while palm oil has been used in Malaysia and Indonesia to produce biodiesel due to the relative abundance of these raw materials in the respective countries (Tan et al. 2010). Other promising sources include non-edible oils such as jatropha (Tiwari et al. 2007; Sahoo and Das 2009), and microalgae and microbial oils some of which have short production cycles and can be produced by fermentation using inexpensive sources, such as CO_2_ or waste water (Mata et al. 2010; Pokoo-Aikins et al. 2010; Schenk et al. 2008). It is therefore necessary that each country or region evaluate the potentials of its available lipid sources for the production of biodiesel. The potentials of mango, neem and shea kernel oils which abound in many (sub)tropical regions of the world for the production of biodiesel fuel have not received much attention from researchers.

The mango tree is grown mostly for its fruits, which contain a sweet pulp, in about 90 countries and is rated among the top 20 agricultural products of the world (FAOSTAT 2013). When mature and ripe, the pulp is mostly eaten in the fresh state as a snack. The peels and the nuts are considered as waste and are thrown away after the fleshy pulp has been consumed.

The neem tree is adapted to hot and dry climates, and is commonly planted in arid and semi-arid areas found in about 78 countries world-wide and is used in a further nine (Förster and Moser 2000). It grows both within its natural range (South Asia) as well as in Sub Saharan Africa, Central and Southern America, the Caribbean, Philippines, and the Middle East.

The shea tree grows mainly in tropical Africa and is today the second most important oil crop in Africa after the palm nut tree, though its potential is not fully exploited. Published data (Maranz et al. 2003) state that at least 500 million production trees are accessible in West Africa, which equates to a total of 2.5 million tonnes of dry kernel per annum (based on 5 kg dry kernel per tree).

Mango (Lakshminarayana et al. 1983), neem (Kaura et al. 1998) and shea kernels contain 7–15, 14–42 and 35–55 % (Bup et al. 2011; Honfo et al. 2013) oils indicating their potentials as a vegetable oil source. While neem oil is not eaten in the production areas, mango and shea oils have been used as cocoa butter equivalents in chocolates and margarine formulations. All three types of oils find use in traditional medicines and cosmetic formulations (Bup et al. 2011; Förster and Moser 2000; Lakshminarayana et al. 1983). However, reports have shown that potential for the production of these non-conventional oils are still largely underexploited. For example only 45 % of total shea kernels produced annually are actually collected and processed (Holtzman 2004). The amounts of neem and mango kernels processed yearly are far less than shea nuts. Consequently it can be stated that these kernels often rot away during the production season and represent instead an environmental concern. At present there are no modern factories in African countries for the processing of mango and neem kernels into oil, which may be due partly to the fact that market demands from the existing users are not strong enough to encourage production. A diversification of the uses of these oils for the production of biodiesel may be a correct measure to encourage their exploitation, which will have important positive effects on the economies of the processors and the countries producing them. Studies on the enzymatic transesterification of these oils, as far as we know, do not exist in the literature.

Response surface methodology has been used by many researchers to establish optimum production conditions for biodiesel (Jeong and Park 2009; Shieh et al. 2003) because of the many advantages the method presents. The objective of this work therefore is to report the lipozyme TL IM catalyzed transesterification yields for biodiesel production from neem, shea and mango oils using response surface methodology and the Doehlert design. The study also reports for the first time ^1^H NMR spectra for mango neem and shea oils.

## Materials and methods

### Materials

Immobilized lipase Lipozyme TL IM from *Rhizomucor miehei* and 99 % methanol used in the experiments were bought from Sigma Aldrich. Ultra-pure water used in the analysis was produced in the laboratory using a Barnstead NANOpure^®^ Diamond™ (Thermo SCIENTIFIC) apparatus. Neem and shea oils were bought from local processors in North Cameroon while mango oil was bought from http://www.Amazon.com. The fatty acid profiles and some physico-chemical properties of the oils used in the analysis are presented in Table 1.

**Table 1:** Fatty acid composition, acid value and moisture content of the oils used for enzymatic transesterification

	Fatty acid composition (%)						Physical properties	
	C16:0	C18:0	C18:1	C18:2	SFA	USFA	Acid value	Moisture content
Mango	15.6	30.2	48.2	6.0	45.8	54.2	1.0 ± 0.1	0.085
Neem	19.6	7.8	51.8	20.8	27.4	72.6	2.4 ± 0.3	0.082
Shea	5.7	41.8	45.6	6.9	47.5	52.5	15.2 ± 0.3	0.230

#### Experimental design

Response surface methodology and the Doehlerts experimental design requiring 42 experiments (21 experiments in duplicate) was used to study the enzyme catalyzed trans-esterification of the three oils into biodiesel. The factors and ranges of the parameters studied were enzyme concentration (3–10 wt% of oil mass), temperature (30–60 °C), quantity of added water (5–15 wt% of oil mass) and time (12–36 h). The solvent/oil mole ratio and the agitation speed were maintained constant at 3:1 and 200 rpm, respectively. The Doehlert matrix used in this study is presented in Table 2.

**Table 2:** Experimental design and conversion yields of mango, neem and shea oils

S/no	Experimental matrix								Responses		
	Coded values				Real values				Conversion yield (%)		
	x_1_	x_2_	x_3_	x_4_	*X*_*1*_	*X*_*2*_	*X*_*3*_	X_4_	Neem	Mango	Shea
1	1	0	0	0	10.00	45.00	10.00	24.00	91.68	92.27	98.77
1	1	0	0	0	10.00	45.00	10.00	24.00	92.71	91.11	99.70
2	−1	0	0	0	3.00	45.00	10.00	24.00	90.51	56.24	58.23
2	−1	0	0	0	3.00	45.00	10.00	24.00	90.77	61.69	60.72
3	0.5	0.866	0	0	8.25	60.00	10.00	24.00	71.74	63.87	99.03
3	0.5	0.866	0	0	8.25	60.00	10.00	24.00	68.98	68.45	99.31
4	−0.5	−0.866	0	0	4.75	30.00	10.00	24.00	94.76	82.69	86.03
4	−0.5	−0.866	0	0	4.75	30.00	10.00	24.00	96.24	85.15	84.15
5	0.5	−0.866	0	0	8.25	30.00	10.00	24.00	93.64	91.38	95.48
5	0.5	−0.866	0	0	8.25	30.00	10.00	24.00	94.34	89.37	94.98
6	−0.5	0.866	0	0	4.75	60.00	10.00	24.00	82.41	48.39	72.86
6	−0.5	0.866	0	0	4.75	60.00	10.00	24.00	86.94	49.38	73.74
7	0.5	0.289	0.816	0	8.25	50.01	15.00	24.00	92.54	91.01	82.70
7	0.5	0.289	0.816	0	8.25	50.01	15.00	24.00	92.53	89.77	85.19
8	−0.5	−0.289	−0.816	0	4.75	39.99	5.00	24.00	89.29	68.70	94.55
8	−0.5	−0.289	−0.816	0	4.75	39.99	5.00	24.00	85.71	70.11	94.70
9	0.5	−0.289	−0.816	0	8.25	39.99	5.00	24.00	93.33	90.82	96.61
9	0.5	−0.289	−0.816	0	8.25	39.99	5.00	24.00	92.52	90.88	97.11
10	0	0.577	−0.816	0	6.50	54.99	5.00	24.00	89.82	77.04	97.07
10	0	0.577	−0.816	0	6.50	54.99	5.00	24.00	88.52	73.64	97.70
11	−0.5	0.289	0.816	0	4.75	50.01	15.00	24.00	97.17	84.99	60.62
11	−0.5	0.289	0.816	0	4.75	50.01	15.00	24.00	93.58	75.32	59.74
12	0	−0.577	0.816	0	6.50	35.01	15.00	24.00	98.68	93.14	81.77
12	0	−0.577	0.816	0	6.50	35.01	15.00	24.00	97.65	92.52	78.24
13	0.5	0.289	0.204	0.791	8.25	50.01	11.25	36.00	97.88	96.30	100.00
13	0.5	0.289	0.204	0.791	8.25	50.01	11.25	36.00	98.90	98.36	99.89
14	−0.5	−0.289	−0.204	−0.791	4.75	39.99	8.75	12.00	93.44	45.32	66.50
14	−0.5	−0.289	−0.204	−0.791	4.75	39.99	8.75	12.00	95.30	47.85	62.75
15	0.5	−0.289	−0.204	−0.791	8.25	39.99	8.75	12.00	95.24	58.58	92.99
15	0.5	−0.289	−0.204	−0.791	8.25	39.99	8.75	12.00	95.46	57.11	97.78
16	0	0.577	−0.204	−0.791	6.50	54.99	8.75	12.00	95.58	71.97	99.78
16	0	0.577	−0.204	−0.791	6.50	54.99	8.75	12.00	95.63	71.15	95.09
18	−0.5	0.289	0.204	0.791	4.75	50.01	11.25	36.00	99.58	88.42	99.65
18	−0.5	0.289	0.204	0.791	4.75	50.01	11.25	36.00	98.52	87.77	99.85
19	0	−0.577	0.204	0.791	6.50	35.01	11.25	36.00	99.79	96.24	99.61
19	0	−0.577	0.204	0.791	6.50	35.01	11.25	36.00	99.80	96.74	99.54
20	0	0	−0.612	0.791	6.50	45.00	6.25	36.00	98.39	97.14	99.54
20	0	0	−0.612	0.791	6.50	45.00	6.25	36.00	95.54	96.33	99.54
21	0	0	0	0	6.50	45.00	10.00	24.00	90.70	97.34	96.62
21	0	0	0	0	6.50	45.00	10.00	24.00	91.43	93.58	98.22
R2									0.808	0.882	0.949
AED (%)\									2.58	5.95	2.85

*X*_*1*_ enzyme concentration, *X*_*2*_ reaction temperature, *X*_*3*_ quantity of added water and *X*_*2*_ reaction time

#### Experimental procedure of the transesterification process

**Fig. 1:**
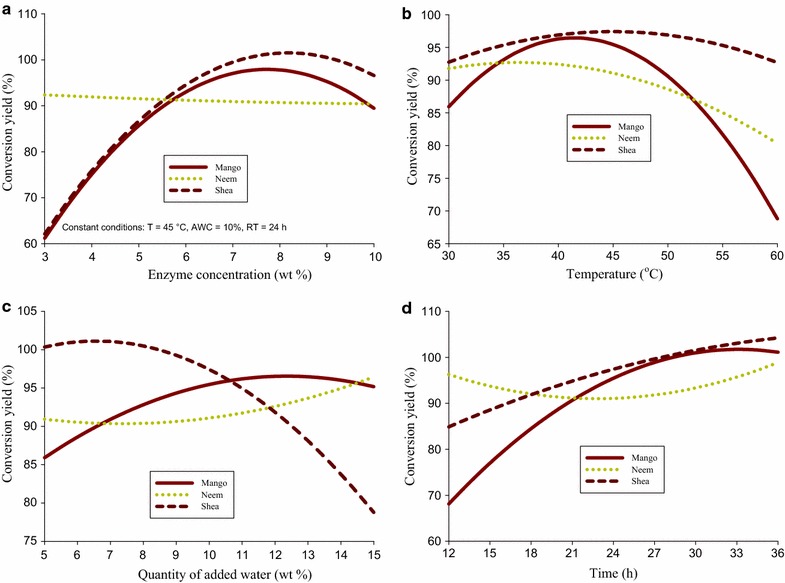
**a** Influence of enzyme concentration on methyl ester yields of the three oils at constant temperature (45 °C), quantity of added water (10 %) and reaction time (24 h). **b** Influence of enzyme concentration (*top*) and temperature (*bottom*) on methyl ester yields of the three oils when the other parameters are maintained constant quantity of added enzyme concentration (6.5 %) water (10 %) and reaction time (24 h). **c** Influence of quantity of added water on methyl ester yields of the three oils at constant enzyme concentration (6.5 %), temperature (45 °C) and reaction time (24 h). **d** Influence of quantity of reaction time on methyl ester yields of the three oils at constant enzyme concentration (6.5 %), temperature (45 °C) and quantity of added water (10 %)

An incubator shaker (C25K New Brunswick Scientific, USA) was used in this study. In each experimental run 5 g of the oil was weighed into a 125 ml conical flask and the calculated amount of enzyme, methanol and water based on the weight of the oil were added in succession to the reaction flask. These were then tightly corked and placed into the incubator shaker and the reaction allowed to run at the required temperature and time as shown in Table 2. From preliminary experiments, addition of 3:1 methanol-oil molar ratio in a single step yielded only about 5 % FAME. Subsequently, methanol was added in three steps to the reaction mixture, with 1/3 of the required amount at 0, 3 and 6 h to achieve a total mole ratio of 3:1. Preliminary experiments (Fig. 1) equally showed that in 2 h almost the entire methanol added had reacted so 3 h addition interval was chosen to ensure a complete reaction. In a solvent system (hexane), reactions yield were lower than those obtained in a solvent free system, therefore all the reactions were carried out in a solvent free system. At the end of each experiment the mixture was filtered through a Whatman filter paper. Samples were then collected and the biodiesel yield analysed by ^1^H NMR spectroscopy (Jin et al. 2007). A total of 42 experiments (21 experiments in duplicates) were carried out for each oil, following the experimental plan based on the Doehlerts experimental design.

### Analysis of biodiesel yield by ^1^H NMR spectroscopy

Samples for ^1^H NMR spectroscopy were prepared by dissolving 0.02–0.05 g of biodiesel produced in 1 ml of chloroform. ^1^H NMR spectra were obtained using a BRUKER 500 MHz AVANCE III instrument with CDCl_3_ as solvent and TMS as an internal standard. ^1^H spectra were recorded with pulse duration of 45 °C and 16 scans, following the procedure described by Gelbard et al. (1995). The percentage conversion (Y) of oil to biodiesel was calculated from the relation (Gelbard et al. 1995; Suganya et al. 2014):

1 $$Y = \frac{{2A_{{CH_{3} }} }}{{3A_{{CH_{2} }} }}$$

A_CH3_ is the integration value of the methoxy protons of the ME (the strong singlet) and A_CH2_ the integration value of the methylene protons. Factors 2 and 3 were derived from the fact that, the methylene carbon possesses two protons, while the alcohol (methanol derived) carbon has three attached protons.

### Statistical analysis

A second order polynomial with interactions represented by Eq. 2 was selected for the analysis. b_0_, b_i_, b_ii_ and b_ij_ are model coefficients for intercept, linear, quadratic and interaction terms, respectively. Coefficients of the model were determined through multiple linear regression analysis using Sigmaplot 12.5 (Systat Software Inc, San Jose, USA).

2 $$y = b_{0} + \mathop \sum \limits_{i = 1}^{3} b_{i} x_{i} + \mathop \sum \limits_{i = 1}^{3} b_{ii} x_{i}^{2} + \mathop \sum \limits_{i = 1}^{2} \mathop \sum \limits_{j = i + 1}^{3} b_{ij} x_{i} x_{j}$$

To optimize the cooking process, the optimum point of Eq. 2 was defined as the point where the first partial derivative of the function equals zero: That is

3 $$\left. {\begin{array}{*{20}l} {\partial y/\partial x_{1} = b_{1} + 2b_{{11}} x_{1} + b_{{12}} x_{2} + b_{{13}} x_{3} } \\ {\partial y/\partial x_{2} = b_{2} + 2b_{{12}} x_{1} + b_{{22}} x_{2} + b_{{23}} x_{3} } \\ {\partial y/\partial x_{3} = b_{3} + 2b_{{13}} x_{1} + b_{{23}} x_{2} + b_{{33}} x_{3} } \\ \end{array} } \right\} = 0$$

The system of equations for each response was then solved using the matrix method using Microsoft Excel (Version 10, Microsoft Corp., Redmond, WA, USA).

#### Validation of models

Two criteria, the regression coefficient (R^2^) and the percentage Absolute Error of Deviation (AED) between experimental and calculated results were used to evaluate the validity of the models. A model was considered valid if R^2^ > 0.7 and/or AED < 10 % (Bup et al. 2012). Regression coefficients were obtained from multiple linear regression analysis carried out on the results using SigmaPlot 12.5 software while AED was calculated from Eq. 4.

4 $$AED\,\left( \% \right) = \frac{100}{p}\sum\limits_{i = 1}^{p} {\left| {\frac{{Y_{\exp } - Y_{\bmod } }}{{Y_{\exp } }}} \right|}$$

where Y_exp_ and Y_mod_ are the values obtained from experiments and from the model, respectively. *p* is the number of points at which measurements were carried out.

## Results

### Modelling of the transesterification process

Validation conditions (R^2^ > 0.8 and AED < 10 %) for the selected second order models obtained from regression analysis were met for all the biodiesels (Table 2). Analysis of variance showed that all the four parameters had significant influences (P < 0.05) on the enzymatic transesterification process of mango, neem and shea oils into biodiesel (Table 3). The validated models were therefore used to generate two dimensional and surface response plots to explain the main and the interaction effects of the factors on the biodiesel conversion yield.

**Table 3:** Statistical analysis for the Doehlert experimental design

	Mango			Neem			Shea		
	*Coeff*	*VMC*	*P* value	*Coeff*	*VMC*	*P* value	*Coeff*	*VMC*	*P* value
b_0_	95.46	37.27	<0.001	91.07	61.86	<0.001	97.42	38.57	<0.001
b_1_	14.14	5.52	<0.001	−0.98	0.67	0.404	17.25	6.83	<0.001
b_2_	−9.89	3.86	<0.001	−6.61	4.49	<0.001	−0.04	0.02	0.973
b_3_	5.68	2.22	0.029	3.37	2.29	0.015	−13.22	5.24	<0.000
b_4_	20.86	8.14	<0.001	1.64	1.12	0.244	12.22	4.84	<0.001
b_11_	−20.13	7.86	0.003	0.35	0.24	0.913	−18.07	7.15	<0.001
b_22_	−24.12	9.42	<0.001	−6.70	4.55	0.044	−6.27	2.48	0.076
b_33_	−7.39	2.89	0.219	3.91	2.65	0.216	−11.81	4.68	0.001
b_44_	−17.32	6.76	0.008	10.37	7.05	0.003	−4.58	1.82	0.186
b_12_	6.25	2.44	0.281	−7.40	5.03	0.02	9.08	3.60	0.009
b_13_	−9.08	3.55	0.165	−2.45	1.66	0.471	9.97	3.95	0.01
b_14_	−1.23	0.48	0.854	2.30	1.56	0.514	−25.21	9.98	<0.001
b_23_	−3.35	1.31	0.602	−4.73	3.21	0.169	−2.61	1.03	0.473
b_24_	−17.45	6.81	0.014	−2.68	1.82	0.447	−7.54	2.99	0.053
b_34_	−3.83	1.50	0.693	2.67	1.82	0.6	17.24	6.83	0.004

### Effect of individual factors on the biodiesel conversion yields of mango, neem and shea oils

Plots of the effect of the individual factors on conversion yields were obtained from the validated equations by maintaining three of the factors constant at their central points and varying the other as a function of the biodiesel conversion. Generally conversion yields ranged from 45.3 to 98.4 %, 69.0 to 99.8 % and 58.2 to 100.0 % for mango, neem and shea oils, respectively.

### Effect of enzyme concentration on biodiesel conversion yield

Table 3 shows that enzyme concentration had a significant effect (p < 0.05) on the biodiesel conversion yields of mango and shea oils. From Fig. 1a it is observed that for mango and shea oils, biodiesel conversion yields increased from about 60 to 97 % and to almost complete conversion, respectively as enzyme concentration increased from 3 to about 7 %. Above 7 % of enzyme, the conversion yields decreased slightly to about 90 % for mango oil but remained almost constant for shea oil. From Table 4, the contribution of enzyme concentration (b_1_ + b_11_) to the value of the conversion yield for neem oil was less than 1 % compared to about 14 % for mango and shea oils, thus illustrating the non-significant effect of this factor on the transesterification of neem oil.

**Table 4:** ANOVA for yield

	*df*	*SS*	*MS*	*F*	*P* value
Mango					
Regression	14	9323.70	665.98	13.80	<0.001
Residual	25	1206.90	48.28		
Total	39	10,530.60			
Neem					
Regression	14	1336.44	95.46	7.15	<0.001
Residual	25	333.99	13.36		
Total	39	1670.42			
Shea					
Regression	14	7095.23	506.80	32.98	<0.001
Residual	25	384.13	15.37		
Total	39	7479.36			

### Effect of temperature on biodiesel conversion yield

For mango oil, conversion yield increased from 85 % at 30 °C to about 95 at 45 °C and then decreased steadily to about 65 % at 60 °C, while for neem oil highest yields were obtained at the beginning of the experiment (<35 °C) followed by a continuous decrease with an increase in reaction temperature (Fig. 1b). While maximum yields for mango and shea biodiesels were obtained at about 45 °C that of neem was at about 35 °C, indicating that enzyme activity may also depend on the type of oil involved in the transesterification process.

### Effect of quantity of added water on biodiesel conversion yield

In this work the quantity of added water was investigated in the range 5–15 % based on the weight of the oil taken for analysis. Conversion yields for all the 3 biodiesels were significantly affected by the quantity of added water. When reaction temperature, time and enzyme concentrations were kept constant at the central point, conversion yields of mango and neem oils increased with the quantity of added water up to a certain value and then remained constant while that of shea oil decreased significantly as the quantity of added water was increased from 5 to 15 % (Fig. 1c).

### Effect of time on biodiesel conversion yield

For mango and shea oils, conversion yields increased respectively from about 70 and 85 % to almost complete conversion as reaction time increased from 12 to 36 h when all other factors were maintained constant at their center points. The linear effect of reaction time on neem biodiesel conversion yield was not significant while the quadratic effect of reaction time was significant explaining the small upward curvature observed on Fig. 1d.

### Interaction effect of studied factors on conversion yields of mango, neem and shea oils

Surface response plots for the interaction effects were generated in SigmaPlot 12.5 to better visualize the combined effects of the factors on yield. The following section describes the interaction effects of two factors while the others were kept constant at the central point on the conversion yield. The interaction effect of the quantity of added water and temperature on yield was not significant for the three biodiesels and has not been discussed.

### Combined effect of enzyme concentration and temperature on conversion yield

**Fig. 2:**
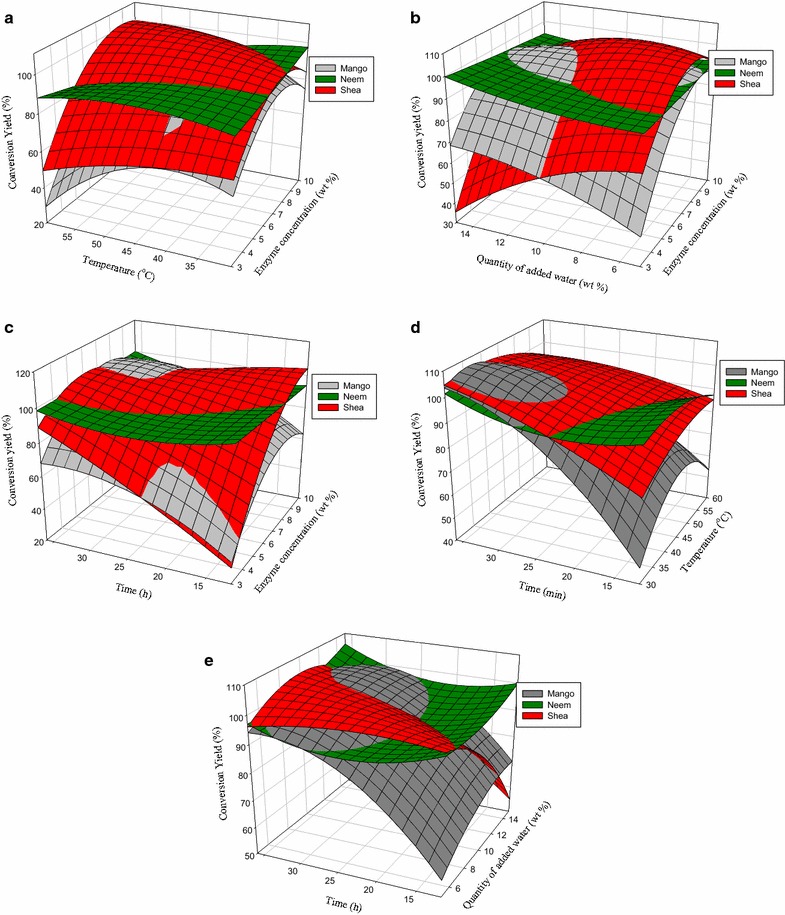
**a** Combined effect of enzyme concentration and temperature on methyl ester yields of the three oils at constant quantity of added water (10 %) and reaction time (24 h). **b** Combined effect of enzyme concentration and quantity of added water on methyl ester yields of the three oils at constant temperature (45 °C) and reaction time (24 h). **c** Combined effect of enzyme concentration and reaction time on methyl ester yields of the three oils at constant temperature (45 °C) and quantity of added water (10 %). **d** Combined effect of temperature and reaction time on methyl ester yields of the three oils at constant enzyme concentration (6.5 %) and quantity of added water (10 %). **e** Combined effect of quantity of added water and reaction time on methyl ester yields of the three oils at constant enzyme concentration (6.5 %) and temperature (45 °C)

Figure 2a gives the combined effect of enzyme concentration and temperature at constant quantity of added water (10 %) and reaction time (24 h). It was significant (p < 0.05) for mango and shea oils but insignificant for neem oil. At low enzyme concentrations, the conversion yields varied only slightly with an increase in temperature for all the 3 oils. As enzyme concentration increased, conversion yields increased with temperature for both mango and shea oils to maximum values and then decreased again as enzyme concentration went above 8 % at temperatures higher than 50 °C. The evolution of the conversion yield of neem oil remains fairly constant irrespective of the variation in temperature and enzyme concentration when the other factors are maintained constant.

### Combined effect of enzyme concentration and quantity of added water on conversion yield

At constant temperature (45 °C) and reaction time (24 h) conversion yields decreased with the quantity of added water irrespective of the enzyme concentrations for shea oil but increased steadily with enzyme concentration especially at lower quantities of added water up to steady values (Fig. 2b). For mango oil, conversion yields increased with the quantity of added water and with enzyme concentration up to steady values. The combined effect of temperature and enzyme concentration was insignificant on neem biodiesel yields; however, these conversions were very high (90–98 %) throughout the experimental period under the explored conditions.

### Combined effect of enzyme concentration and reaction time on conversion yield

The variation of enzyme concentration and reaction time on conversion yield is shown in Fig. 2c. At lower enzyme concentration, initial conversion yields for mango oil were greater than that of neem up to about 20 h reaction time. After 20 h that of shea oil was greater than that of mango oil. At high enzyme concentrations conversion yields increased with reaction time and attained steady values for both mango and shea oils. Conversion yields were higher for shea oils compared to mango and neem oils. Variation of conversion yields for neem oils under these conditions was not significant. Maximum yields for shea oils were obtained at the highest enzyme concentration. At prolonged reaction times, conversion yields decreased probably due to aggregation of enzymes after long reaction times. In fact it was visually observed that enzymes aggregated and settled at the bottom of the reaction flask for most of the experimental runs that lasted more than 24 h.

### Combined effect of temperature and reaction time on conversion yield

When the enzyme concentration and quantity of added water were held constant at their center points, only the variation of the conversion yield of mango oil was significant (Table 2). For mango oil, at temperatures lower than 40 °C, conversion yield increased from less than 50 % to close to 100 % as reaction time increased from 12 to 36 h (Fig. 2d). As temperatures increased above 40 °C, mango oil conversion yields decreased significantly and were lowest at 60 °C. There was however no significant difference on shea and neem oil conversion yields at higher temperatures.

### Combined effect of quantity of added water and reaction time on conversion yield

Figure 2e shows the variation of conversion yield for the 3 different oils as a function of quantity of added water and reaction time at constant enzyme concentration (6.5 %) and reaction temperature (45 °C). The interaction effect of reaction time and quantity of added water on conversion yields of mango and neem oils was not significant, contributing less than 2 % to the value of the conversion yields of both biodiesels. On the other hand, the combined effect of the two factors (b_34_) had a significant contribution to the conversion yield of shea oil. At shorter reaction times, conversion yields for shea oil decreased sharply with an increase in the quantity of added water and then remained fairly constant as reaction proceeded (Fig. 2e). At quantities of added water greater than 10 % conversion yields for shea oil again decreased sharply with prolonged reaction times. Though the combined effect of these factors had no significant effect on mango yields, it can be observed from Fig. 2e that, at shorter reaction times, its conversion yields increased with an increase in the quantity of added water. This increase became more noticeable at longer reaction times. The statistically insignificant yet noticeable effect of the interaction effect of reaction time and temperature on the conversion yield of mango is due to the high contribution of the individual effect of reaction time (b_4_) which was greater than 8 %. Note that b_4_ had a significant effect on mango oil conversion yields. Again one notices the different behaviors of the different oils under similar reaction conditions.

### Optimization of the enzymatic transesterification processes

Optimizations of the independent parameters gave the following optimum conditions: enzyme concentration 7.26 %, temperature 36.6 °C, quantity of added water 10.9 % and reaction time of 36.4 h for the production of mango oil ME. Corresponding values for neem and shea oils ME were 7.19 %, 45.65 °C, 8.43 % and 25.08 h and 4.43 %, 45.65 °C, 6.21 % and 25.08 h, respectively. ME yields calculated using the validated second order models showed that, under these conditions, a 100 % conversion of the oils into biodiesel was achieved. Verification experiments conducted at these calculated optimum points reached conversion yields of 98.09 ± 0.96, 98.48 ± 1.62 and 99.25 ± 0.35 % for mango, neem and shea oils which were not significantly different from the calculated yields, indicating that the developed models are adequate for use in describing enzymatic transesterification of these oils.

## Discussion

### Effect of individual factors on the biodiesel conversion yields

Decrease in biodiesel conversion yields at high temperatures has been linked to the aggregation of enzymes at such temperatures. A similar observation was made in the trans-esterification of corn oil using lipozyme TL IM as catalyst (Wang et al. 2008). The variation of the conversion yield with enzyme concentration at constant temperature, time and quantity of added water, was insignificant (P > 0.05) for neem oil and varied only from 90 to 92 % indicating that low enzyme concentrations can be used to achieve high biodiesel yields. This will reduce the cost of the production process.

Transesterification temperature had a significant effect on the yields of mango and neem biodiesels but not on that of shea biodiesel. Reaction rate increases with temperature and reaction time due to the reduction of viscosity of the oil. This is favorable to increase the solubility of the oil in methanol and improve the contact between oil and methanol molecules, thereby reaching a better yield of ME (Suganya et al. 2013). Decrease in conversion yields after a certain temperature indicates the optimum temperature for enzyme activity after which the enzymes are denatured and can therefore not take part in the reaction. Several studies have indicated that in enzymatic catalysis each enzyme has an optimum temperature over which biodiesel yields are highest and this may depend on the type of oil used in the analysis (Bajaj et al. 2010; Dizge and Keskinler 2008).

### Interaction effect of factors studied on conversion yields

For the combine effect of enzyme concentration and temperature on conversion yields it was observed that higher values of these parameters decrease conversion yields. The decrease in conversion yields above a certain enzyme concentration and temperature is attributed to the agglomeration and heat denaturation of the enzymes which both lower enzyme activity (Bajaj et al. 2010; Dizge and Keskinler 2008).

The presence of sufficient quantities of water in the reaction system can enhance the efficiency of lipase catalyzed reactions, while insufficient amounts of water in the reaction mixture can cause inactivation of lipase (Kaieda et al. 2001). Water addition to the reaction system may also reduce the resistance to mass transfer which results from the accumulation of glycerol produced during the reaction (Tran et al. 2012). However the amount of added water should be controlled as the presence of excess water also has negative consequences on the reaction yield. The quantity of added water should therefore be monitored carefully in order to determine the optimum quantity required for highest yields. According to Miller et al. (1988) and Yamane (1987), it is important to protect the water surrounding lipases for optimal conformation of the enzyme, and removal of the water can lead to both reversible, but mainly irreversible, changes in the protein structure. Previous studies have shown that the optimum quantity of added water may depend among other factors on the type of enzyme and the substrate (Kaieda et al. 2001). In this work it was observed that, in addition to the introduced factors (EC, T, AWC and RT) the quantity of water originally present in the oil before transesterification may play an important role in the process. For example the initial quantity of water (0.229 ± 0.02 %) present in shea oil was significantly higher than that of mango (0.082 ± 0.01) and neem (0.084 ± 0.01) oils, respectively. This may explain the observed decrease of conversion yields on addition of water to shea oils, as addition of water above a certain level favors hydrolysis of the triglycerides instead of the transesterification reaction (Fjerbaek et al. 2009). At higher enzyme concentration (>8 %), the conversion yields remained fairly constant up to quantities of added water of 10 %. When quantity of added water surpassed 10 % the conversion yield again decreased. We infer from this behavior that as the concentration of enzyme increases, the negative effect of water on conversion yield is overpowered by increased enzyme activity but with more and more water added to the system, enzyme activity is again reduced.

Considering the combined effect of temperature and reaction time the observed decreased conversion yields of mango oil at higher temperatures was linked to the denaturation of the enzymes at higher temperatures as earlier adduced. There was however no significant difference on neem and shea yields as a result of the interaction effect of reaction time and temperature even at higher temperatures. This difference in the behavior of the conversion yields of the oils indicate that the lipozyme enzyme used in this work could be more tolerant to high temperatures in neem and shea oils compared to mango oils. It may be possible that mango oil may have some specific compound that may denature/deactivate the enzyme. This assertion requires further analysis. Again under these conditions shea conversion yields were for most of the times higher than those obtained with mango and neem oils.

### Optimization

It was observed that optimum points for the transesterification of these oils under the same conditions differ between the oils. For example optimum operation temperature for mango oil was 36.6 °C and therefore required a longer time of 36 h to attain complete conversion. Lipozyme was more tolerant to higher temperatures in neem and shea oils with a consequent reduction of the reaction time (25 h) required to attain 100 % conversion. Optimum conditions were reported elsewhere for biodiesel production using immobilized lipozymes from *Mucor miehei* for oil/ethanol molar ratio, temperature, added water content, and amount of enzyme of 1:3, 50 °C, 0 % (vol/vol), and 0.4 g of Lipozyme per 5.7 mmol of sun flower oils, respectively (Selmi and Thomas 1998). Shieh et al. (2003) also reported that optimum synthesis conditions giving 92.2 % weight conversion of soybean methyl esters using lipase from *Mucor mieher* were: reaction time 6.3 h, temperature 36.5 °C, enzyme amount 0.9 BAUN (Batch Acidolysis Units NOVO), substrate molar ratio 3.4:1, and added water 5.8 %.

These results point to the fact that optimum conditions for transesterification vary with a variety of factors. In this work, since the three oils were processed under the same conditions using lipase from *Mucor miehei*, the differences observed in the behaviors of the yields of ME of the three different oils as seen in the preceding sections could be linked to one or a combination of composition, initial moisture content and the percentage of FFA present in the oil. Fatty acid compositions of the different oils used in the analysis are presented in Table 1 which shows that neem oil is composed of more than 70 % unsaturated fatty acids while shea and mango oils were composed of almost equal proportion of saturated and unsaturated fatty acids. The polyunsaturated nature of the neem oil can explain to a certain degree the reduced variation of the conversion yields of neem ME esters under most of the reaction conditions, since the oil remained in the liquid phase for the most part under these experimental conditions. In some cases especially at low temperatures, shea and mango oils existed first in the solid phase before dissolution with the progression of the reaction and this probably could have led to the observed significant variations of mango and shea biodiesels yields under the explained conditions.

Note that maximum yields were highest for shea oils compared to neem and mango oils. This was attributed to the high levels of free fatty acids (15.18 ± 0.25 %) in shea oil compared to 0.95 ± 0.05 and 2.42 ± 0.29 % for mango and neem oils. Table 2 clearly indicates that under the same conditions for most of the experiments the conversion yields varied in direct proportion with the acid value of the oil. That is, conversion yields were highest for shea, followed by neem and mango. One advantage of enzyme catalyzed transesterification reactions is that oils with high FFA can be easily converted to MEs without prior treatment as done in chemical catalysis, probably because no energy needs to be expended by the enzyme-based process to separate the FFA from the glycerol backbone (Lai et al. 2005).

Some quality parameters of the biodiesel determined following the ASTM methods (Table 5) all met the ASTM biodiesel standards.

**Table 5:** Properties of fatty acid methyl esters as compared to international standard requirements of ASTM

Property	Units	FAME value			Required by ASTM
		Mango	Neem	Shea	
Kinetic viscosity at 40 °C	mm^2^ s^−1^	3.25	2.668 ± 0.027	5.404	D 445 (1.9–6.0)
Density at 40 °C	kg m^−3^	0.870 ± 0.000	0.858 ± 0.002	0.865 ± 0.006	
Acid value	mgKOH g^−1^	0.33 ± 0.008	0.18 ± 0.03	0.37 ± 0.002	D 664 (Max. 0.5)
Cetane number		57.83	64.43	64.75	D 613 (47 minimum)
Water content	% volume	0.023 ± 0.008	0.044 ± 0.007	0.047 ± 0.005	D 2709 (0.050 %)
Cloud point	°C	17–18	15–17	19–20	ASTM D6751 (−3 to 12)
Pour point	°C	13	12	13	ASTM D6751 (−15 to 10)

From the foregoing, it can be concluded that under the respective optimum conditions of enzyme concentration, temperature, quantity of added water and reaction time of 7.26 %, 36.61 °C, 10.90 % and 36.42 h for mango oil, 7.19 %, 45.65 °C, 8.43 % and 25.08 h for neem oil and 4.43 %, 45.65 °C, 6.21 % and 25.08 h there was complete conversion of the oils into biodiesel. Under similar conditions shea oil produced more biodiesel compared to mango and neem oils. Optimum processing conditions differ for each oil and therefore emphasize the need for such studies on each oilseed to establish precise parameters for appropriate scale up and industrial applications. The use of immobilized lipase from *Mucor miehei* is encouraged because apart from efficiently catalyzing the reaction, it is cheap and can be reused up to 10 times without significant loss of activity (Krishna et al. 2001).
